# Draft Genome Sequence of the Obligately Alkaliphilic Sulfate-Reducing Bacterium *Desulfonatronum thiodismutans* Strain MLF1

**DOI:** 10.1128/genomeA.00741-14

**Published:** 2014-07-31

**Authors:** Denis Trubitsyn, Corey Geurink, Elena Pikuta, Christopher T. Lefèvre, W. Michael McShan, Allison F. Gillaspy, Dennis A. Bazylinski

**Affiliations:** a School of Life Sciences, University of Nevada at Las Vegas, Las Vegas, Nevada, USA; b Department of Mathematical, Computer and Natural Sciences, Athens State University, Athens, Alabama, USA; c CEA/CNRS/Aix-Marseille Université, Biologie Végétale et Microbiologie Environnementales, Laboratoire de Bioénergétique Cellulaire, Saint Paul, France lez Durance, France; d Department of Pharmaceutical Sciences, University of Oklahoma Health Sciences Center, Oklahoma City, Oklahoma, USA; e The Laboratory for Molecular Biology and Cytometry Research/Genomics Division, University of Oklahoma Health Sciences Center, Oklahoma City, Oklahoma, USA

## Abstract

*Desulfonatronum thiodismutans* strain MLF1, an alkaliphilic bacterium capable of sulfate reduction, was isolated from Mono Lake, California. Here we report the 3.92-Mb draft genome sequence comprising 34 contigs and some results of its automated annotation. These data will improve our knowledge of mechanisms by which bacteria withstand extreme environments.

## GENOME ANNOUNCEMENT

Highly alkaline aquatic environments (pH >9.0) are relatively uncommon on Earth and include soda lakes (some of which are hypersaline) and some hydrothermal springs. Despite the hostile conditions in soda lakes, they are among the most productive aquatic environments on the planet (1, 2). Prokaryotes whose growth and survival depends on high pH are termed alkaliphiles (2). Alkaliphilic bacteria face the major problem of maintaining an internal pH at a level not higher than approximately pH 8.5 and use a number of strategies to mitigate this problem (2). The enzymes of alkaliphiles have been used in a variety of industrial applications (e.g., alkaline proteases are widely used in the detergent industry) (3). A good example of an obligate alkaliphile is *Desulfonatronum thiodismutans*, an anaerobic sulfate-reducing bacterium isolated from Mono Lake, California (4). This species grows chemolithoautotrophically or chemolithoheterotrophically on hydrogen gas. It appears to possess a high affinity to molecular hydrogen and in natural ecosystems it is found in tight symbiotic relations with primary anaerobes (e.g., saccharolytic spirochetes) that produce hydrogen as a product of fermentation (4).

The genome of *D. thiodismutans* strain MLF1 was sequenced with an Illumina MiSeq instrument using paired-end 150-bp chemistry. Sequencing resulted in 5,840,423 mapped reads with a mean length of 190. 5,320,956 reads were in aligned pairs, with a mean distance of 241. Paired reads were assembled *de novo* using CLC Genomics Workbench, and contigs with a length of >2 kb were considered for further analysis. The genome size for *D. thiodismutans* was previously estimated to be ~1.6·× 10^9^ Da (~2.88 Mb) (4). Based on this genome size, our sequencing coverage was ~280-fold. The *de novo* assembly resulted in a draft genome comprising 34 contigs with a total of 3,923,724 bp. The mean G+C content determined by genome sequencing is 58.74% (compared to 59.0 ± 0.1 mol% as determined by high-pressure liquid chromatography [HPLC]) (4, 5). Automated annotation by RAST (6) generated 3,737 predicted coding sequences, including 1,329 hypothetical proteins and 51 total RNAs. Our results are similar to those of *Desulfonatronum lacustre* DSM 10312, whose genome size was determined to be 3,761,451 bp (GenBank accession number JAFE01000000). Although both species share many phenotypic similarities, including the dissimilatory sulfate reduction and the requirement for high pH for growth, the genome of *D. thiodismutans* contains 78 unique genes, based on a function-based comparison in RAST, while that of *D. lacustre* DSM 10312 contains 74 unique genes. For example, *D. thiodismutans* possesses genes involved in capsule formation and for encoding a beta-lactamase.

Our genomic study of *D. thiodismutans* should provide valuable information on the evolution and mechanisms of alkalitolerance, lithotrophy, and the inorganic fermentation of thiosulfates and sulfites. In addition, some magnetotactic bacteria appear to be strains of *D. thiodismutans* (7, 8). Genome comparisons between these and the type strain might provide evidence on how magnetosome genes are acquired (e.g., by horizontal gene transfer) or lost.

### Nucleotide sequence accession number.

This whole-genome shotgun project has been deposited in DDBJ/ENA/GenBank under the accession number JPIK00000000.

## References

[1] Jones BE Grant WD Duckworth AW Owenson GG 1998 Microbial diversity of soda lakes. Extremophiles 2:191–200. 10.1007/s007920050060 9783165

[2] Jones BE Grant WD Collins NC Mwatha WE 1994 Alkaliphiles: diversity and identification, p 195–230 *In* Priest FG Ramos-Cormenzana A Tindall BJ (ed), Bacterial diversity and systematics. Plenum Press, New York, NY.

[3] Horikoshi K 1999 Alkaliphiles: some applications of their products for biotechnology. Microbiol. Mol. Biol. Rev. 63:735–750 10585964 10.1128/mmbr.63.4.735-750.1999 PMC98975

[4] Pikuta EV Hoover RB Bej AK Marsic D Whitman WB Cleland D Krader P 2003 *Desulfonatronum thiodismutans* sp. nov., a novel alkaliphilic, sulfate-reducing bacterium capable of lithoautotrophic growth. Int. J. Syst. Evol. Microbiol. 53:1327–1332. 10.1099/ijs.0.02598-0 13130014

[5] Whitman WB 2006 Error in G+C calculations. Int. J. Syst. Evol. Microbiol. 56:1177. 10.1099/ijs.0.64374-0

[6] Overbeek R Olson R Pusch GD Olsen GJ Davis JJ Disz T Edwards RA Gerdes S Parrello B Shukla M Vonstein V Wattam AR Xia F Stevens R 2014 The SEED and the rapid annotation of microbial genomes using subsystems technology (RAST). Nucleic Acids Res. 42:D206–D214. 10.1093/nar/gkt1226 24293654 PMC3965101

[7] Lefèvre CT Frankel RB Pósfai M Prozorov T Bazylinski DA 2011 Isolation of obligately alkaliphilic magnetotactic bacteria from extremely alkaline environments. Environ. Microbiol. 13:2342–2350. 10.1111/j.1462-2920.2011.02505.x 21605309

[8] Lefèvre CT Trubitsyn D Abreu F Kolinko S Jogler C de Almeida LG de Vasconcelos AT Kube M Reinhardt R Lins U Pignol D Schüler D Bazylinski DA Ginet N 2014 Comparative genomic analysis of magnetotactic bacteria from the *Deltaproteobacteria* provides new insights into magnetite and greigite magnetosome genes required for magnetotaxis. Environ. Microbiol. 15:2712–2735. 10.1111/1462-2920.12128 23607663

